# Age Differences in Online News Consumption and Online Political Expression in the United States, United Kingdom, and France

**DOI:** 10.1177/19401612211060271

**Published:** 2021-12-20

**Authors:** Shelley Boulianne, Adam Shehata

**Affiliations:** 1 Department of Sociology, MacEwan University, Edmonton, Alberta, Canada; 2 Journalism Media and Communication JMG, University of Gothenburg, Göteborg, Sweden

**Keywords:** youth, online news, political expression, cross-national

## Abstract

Younger and older generations are differently motivated in relation to news consumption and online political expression. In this paper, we suggest that different modes of citizenship characterize younger and older generations. To test the differential role of political interest in news consumption and online political expression, we use a survey of 3,210 people from the United States, 3,043 from the United Kingdom, and 3,031 from France. Our findings suggest that young citizens are more frequent users of online news overall and that the rank order of different news activities replicates cross-nationally. The frequency of online political expression is negatively related to age, with older people less likely to post online. Age moderates the relationship between political interest and news consumption as well as news consumption and online political expression. The correlations of these sets of variables are stronger for younger respondents compared to older respondents. These findings hold across the three countries under study. We explain these patterns in terms of changing citizenship norms and discuss the implications for democracy.

Understanding generational differences in news media use and political engagement is fundamental for democracy. Extensive research suggests that age is the most important socio-demographic factor behind news consumption in today’s high-choice media environments. On the one hand, younger generations largely reject the traditional news media sources preferred by older people (Newman et al. 2020), creating a generational divide in traditional news consumption. On the other hand, youth are much more likely to get their news from online sources in general—and social media in particular (Ohme 2019), where they are also incidentally exposed to news (Gil de Zúñiga et al. 2020). The affordances of social media allow them to not just read news, but to share and comment on stories as well (Andersen et al. 2020). As such, young adults are sometimes described as a distinctive cohort socialized to use new media platforms for news and political engagement (Andersen et al. 2020; Bennett et al. 2011).

These generational differences have been conceptualized as reflecting changing modes of citizenship across Western democracies (Bennett 2008; Dalton 2008). The theoretical distinction between *self-actualizing* citizenship (AC) and *dutiful* citizenship (DC) aims to capture not only generational differences in media use but also how citizens relate to and engage in politics more generally. A key factor distinguishing these modes of citizenship is *political interest*: whether engagement with news and politics is driven by a personal intrinsic motivation (AC) or by an extrinsic sense of duty or importance (DC). While young citizens are more cause-oriented, motivated by self-actualizing goals as they navigate the digital media environment and engage in politics, older generations rely more on socialized habits oriented toward institutional politics and traditional news media. In addition, personal motivation and political interest, in particular, are currently seen as becoming increasingly important for understanding news consumption and political behavior in today's high-choice media environments (Prior 2007; Strömbäck et al. 2013).

This study aims to test a specific argument derived from this literature that political interest is a stronger covariate of news consumption among young citizens, compared to older generations (H1). We base this on scholarship about changing norms of citizenship (Bennett 2008; Lane 2020; Ohme 2019; Shehata et al. 2016) as well as research on the role of political interest (Prior 2007; Thorson 2015). We further test whether the correlation between news consumption and online political expression is stronger for young people compared to older people (H2), again reflecting on scholarship about generational differences and citizenship norms. Based on the theory, we expect a specific causal structure, but our data are correlational and cannot test causality. We rely on existing longitudinal studies to base our claims about causal flow (Boulianne 2011; Kruikemeier and Shehata 2017; Möller et al. 2018a; Shehata 2016; Thorson et al. 2018). We use cross-national data from three Western democracies: the United States, the United Kingdom, and France. We expect these relationships to resonate across different political contexts and that generational differences are more profound than cross-national differences.

## Online News Consumption

The presence of generational gaps in news consumption is a consistent theme in research on news media use. Questions relating to the nature, size, and causes of these gaps are widely discussed. Generational approaches typically explain differences in news consumption in terms of both changing norms of citizenship and media-technological developments.

Classic notions of citizenship particularly emphasize news consumption as critical for politically informed citizens (Delli Carpini and Keeter 1996; Kligler-Vilenchik 2017; Schudson 1998; Ytre-Arne and Moe 2018). For instance, Schudson (1998) outlines the evolution of civic norms from the “informed citizen” to the “monitorial citizen.” The monitorial citizen pays some attention to the news, being on alert for key events that may require their attention, such as natural disasters or violent conflicts (Schudson 1998; Ytre-Arne and Moe 2018). As noted by Zaller (2003: 110) “news should provide information in the manner of attention-catching ‘burglar alarms’ about acute problems, rather than ‘police patrols’ over vast areas that pose no immediate problems.” Others argue that news consumption is important for the ongoing monitoring of government performance (Toff and Kalogeropoulos 2020; Zaller 2003): Citizens use the news media to monitor current events and government performance and act when necessary.

As such, older people's greater news media use may partly reflect a generational difference in what it means to be a good citizen. For older generations, being informed is critical to being a good citizen; however, research on citizenship norms tends to focus on youth, which inhibits the testing of generational differences in citizenship norms (Lane 2020; Shehata et al. 2016). Lower news consumption among young citizens is therefore typically framed as a problem from an informed and DC perspective. If younger people do not view being informed as an important component of being a good citizen, this can manifest in lower levels of news consumption but also a complete avoidance of news (Edgerly et al. 2018; Möller et al. 2018a; Shehata 2016; Toff and Kalogeropoulos 2020). Without established news habits, young people may wait for the news to find them (Edgerly 2017; Gil de Zúñiga et al. 2020).

At the same time, the opportunities to stay informed are exponentially greater in today's digital media environment than in prior eras. Because news is readily available to all, we might actually expect few differences in online news consumption. Yet, some argue that while younger generations were raised in a high-choice media environment, they did not develop a preference for news; instead, the ample choices led to being overwhelmed to the point of foregoing any news (Edgerly 2017; Edgerly et al. 2018). Also, if self-actualizing norms of citizenship dominate among young citizens, rather than dutiful norms, developing such personal content preferences and motivations should be more decisive for whether young citizens follow news.

When young people do access news, it tends to be filtered through social media. The 2020 Digital News Report documents that younger people prefer digital platforms for news consumption, compared to print and broadcast (Newman et al. 2020). Among 18- to 24-year-olds, 38% identified social media as their main method of news consumption in the past week, compared to 26% for other age groups (see page 11). The report also notes cross-national differences in online news consumption. In particular, online news consumption is more popular in the United Kingdom (77%) and the United States (72%) compared to France (66%). However, we do not know if these cross-national differences are consistent across age groups (Newman et al. 2020). The differences among these three countries are quite small compared to the news consumption differences revealed in an analysis of 35 countries (Toff and Kalogeropoulos 2020). However, Toff and Kalogeropoulos (2020) show the United States has the highest news avoidance of the three countries, with the United Kingdom having the lowest and France in the middle. These countries all fit with Toff and Kalogeropoulos’ (2020: 367) “cultures of news consumption” given their high degree of press freedom and political stability.

Taken together, debates continue about the size of the generational differences in news consumption. In particular, survey research has been criticized for exaggerating the generational gap, whereas web tracking data suggest the generational differences are smaller than implied by survey research (Mangold et al. 2021; Scharkow et al. 2020). This digital trace research also suggests the generational gaps in patterns of news consumption are similar in the United States and Germany (Mangold et al. 2021).

*(RQ1) What are the age differences in online news consumption?*

## Political Interest versus Civic Duty

News consumption and political participation both depend on a combination of opportunities, motivations, and abilities (OMA; Andersen et al. 2020; Delli Carpini and Keeter 1996; Strömbäck et al. 2013). Several studies suggest that *political interest* is one of the most important personal motivations explaining news media use and various forms of political engagement (Prior 2010; Shehata and Amnå 2019). This may particularly be the case in a high-choice media environment where citizens can select among a seemingly endless amount of media content and modes of participation based on their preferences and interests. Understanding the role of political interest is therefore crucial for a broader understanding of engagement in political and civic life in contemporary democracies.

At the same time, political interest is a form of “involvement” distinct from political behavior (Andersen et al. 2020). Conceptualized as an intrinsic motivation to engage with political matters, political interest has been defined as “a citizen's willingness to pay attention to political phenomena at the possible expense of other topics” (Lupia and Philpot 2005: 1122; see also Möller et al. 2018b; Russo and Stattin 2017; van Deth 1990). Irrespective of whether political interest is conceptualized as a psychological state or a trait (Hidi and Renninger 2006; Prior 2010), it is strongly linked to various forms of political activities.

The importance of political interest as a predictor of news consumption and political behavior may, however, vary across groups of citizens. More specifically, we argue that generational differences matter. Building upon the work by Thorson (2015) as well as research on generational modes of citizenship (Bennett 2008; Dalton 2008), we suggest that political interest is a more important motivational factor among younger than older groups of citizens. The distinction between AC and DC ultimately reflects a generational difference in norms of good citizenship and motivations for engaging in politics. While the two modes of citizenship are distinct ideal types, Bennett (2008) argues that AC orientations are more prevalent among younger generations, while DC orientations are more common among older citizens (see also Dalton 2008; Ohme 2019; Shehata et al. 2016).

Dutiful citizens rely primarily on a sense of duty, or perceived importance, as a motivation to engage in politics. DC participation is mainly oriented toward institutional politics. Self-actualizing citizens, however, are less motivated by duty, relying instead on a “higher sense of individual purpose” (Bennett 2008: 14). Vromen et al. (2016) explain that “new conceptions [of citizenship] portray young citizens as floating around in a fluidity of personalised impulses to engage or not” (p. 514). Participation is less habitual, more detached from institutional politics, and mainly cause- or issue-oriented (Shehata and Amnå 2019; Vromen et al. 2016). In interviews with 40 American youth, Thorson (2015: 19) identifies a recurring theme—that political participation is a lifestyle choice driven by personal interest rather than a duty:

Political interest has always been an important motivator of participation, but these data suggest it is increasingly the key resource differentiating those who play with and innovate and demonstrate the happy side of political *Do It Yourself* (D.I.Y.) and those who exclude themselves through various routes.

Thus, we argue that political interest is a proxy measure for this “individual purpose” or “impulse” to participate. This has implications for what role political interest plays among younger and older citizens—in ways that affect patterns of both news consumption and political engagement. Among younger generations, having a strong personal interest in politics should be more decisive for whether they engage with news and if they act politically on the news stories they consume. Having a personal interest in politics should, however, matter less among older generations, who are guided more by a sense of duty as well as by socialized habits in relation to both news consumption and political participation.

With respect to media use, studies suggest the relationship between political interest and news consumption has increased over recent decades (Strömbäck et al. 2013). These trends could imply generational differences in the importance of political interest for news media use. This interpretation is supported by a recent Swedish study showing the relationship between political interest and news consumption is substantially stronger among the youngest age group (16–29 years old) than among the older groups (30–49, 50–64, and 65–85 years) (Andersson 2019). In addition, the generational socialization of media habits plays an important role here. Research suggests that citizens’ news consumption habits take shape in formative years and remain relatively stable over the life cycle (Ghersetti and Westlund 2018; Westlund and Weibull 2013). Growing up in a low-choice legacy media environment during the 1950s, 1960s, 1970s, or 1980s is completely different than growing up in today's high-choice internet era. As noted by Westlund and Weibull (2013: 167),

when a generation has grown old they have already developed strong media habits, which change slowly, whereas when individuals are young there is greater responsiveness to emergent news media that has to do with them being in a formative phase in life. During this period they are more open to trying new media and domesticating these into their lives.

Accordingly, early formed news consumption habits among older generations imply that political interest should be a less important factor for whether they follow news or not. Given the universality of these long-term media environmental developments, we expect this relationship to hold across countries.

**H1:** The positive relationship between political interest and news consumption will be stronger for younger (vs. older) respondents.

## Political Expression on Social Media

As noted above, different models of citizenship relate not only to potential generational differences in news consumption but to forms of political participation. In addition to identifying “being informed” as a core premise (Delli Carpini and Keeter 1996; Kligler-Vilenchik 2017), the dutiful model is mainly tied to institutional politics. Voting and other government-centered activities are crucial (Bennett 2008; Shehata et al. 2016), along with joining political parties. The dominant form of communication is a mass media-centered, one-way, top-down broadcast model of information dissemination (Bennett 2008; Shehata et al. 2016). As argued by Bennett (2008: 19), “The informed citizen is supposed to take abstract, impartial information and then decide how to apply it. This model simply doesn't work for AC citizens. They are skeptical of official versions of events. They prefer to help assemble and deliberate about information.”

In the dutiful model of citizenship, political expression is defined in relation to contacting officials, rather than social expression (Bennett et al. 2011). However, AC values self-expression (Bennett 2008; Kligler-Vilenchik 2017; Lane 2020; Shehata et al. 2016). Digital media are crucial for these alternative models of citizenship (Kligler-Vilenchik 2017). These models differ in terms of whether new forms of citizenship **depend** on digital media, that is, participatory civics (Kligler-Vilenchik 2017), as opposed to digital media simply being a **tool** that participatory communities use (Jenkins et al. 2016). However, these models converge on the idea about the importance of “interactive, creative performative, and co-production dimension of online media use” (Shehata et al. 2016: 1146).

Self-actualizing models stress creative expression online, such as writing blogs and creating political videos (Kligler-Vilenchik 2017; Lane 2020) as well as sharing political information on social media and adding comments to news websites. Quite simply, actualizing models of citizenship demonstrate the “lines between content consumption and production [are] blurred” (Bennett et al. 2011: 840). In addition, there is a strong desire to share content with peer networks (Bennett et al. 2011). Because of these correlated citizenship norms (informed plus expression), we might see a stronger correlation between online news consumption and political expression for young adults compared to older adults. For young adults with a strong interest in politics, consuming news is synonymous with commenting on and sharing the news. Andersen et al. (2020: 25; see also Thorson, 2015) write,

drawing on the life-cycle perspective, in their formative stages of life, young citizens are typically more curious and search for ways to express or promote themselves, which may influence both their search for political information and their reactions to this information.

Using the Digital News Report, Kalogeropoulos (2017) finds American respondents (41%) are more likely to report sharing the news compared to French (32%) and British respondents (22%). These cross-national differences are also stable over time (Kalogeropoulos 2017). Thus, although we might expect some cross-national differences in online political expression based on these findings, our purpose is not to examine differences in rates of participation. Our aim is rather to understand the factors behind the online political expression. As such, we expect similarity in the role of age, political interest, and online news consumption in relation to the online political expression: online news consumption should be strongly connected to political expression in a similar manner across all countries. Thus, we do not believe that the underlying causal mechanisms are different in each country. Where that regression line begins and ends may differ by country (as the descriptive research shows), but we expect the slope to be consistent across countries.

**Figure 3.**
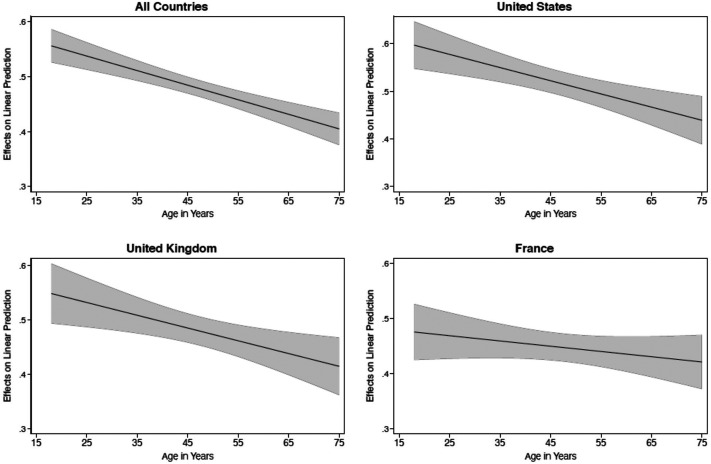
The marginal effect of political interest on online news use across age groups.

*Note.* The marginal effect of political interest on online news use and 95% confidence levels, based on the regression models in Table 1.

Using an American sample of young adults, Lane (2020) finds that news consumption and political interest have the largest impacts on political expression on social media (also see Keating and Melis 2017). Lane (2020) also finds that expressive norms, that is, perceived importance of expressing oneself on social media, were the least important norms examined (see Figure 3, p. 271). In contrast, obedient norms, that is, obeying laws, scored higher in importance. Furthermore, these expressive norms were modestly related to the political expression on social media (Lane 2020). As such, instead of asking about the perceived importance of an activity for being a good citizen, we focus on news consumption as a predictor of online political expression; we examine how this relationship differs for different age groups.

**Table 1.** Political Interest on Online News (OLS Estimates).

	All countries	United States	United Kingdom	France
Political interest	0.60*** (0.02)	0.65*** (0.04)	0.59*** (0.04)	0.49*** (0.04)
Age (18–75 years)	−0.00** (0.00)	−0.01** (0.00)	−0.01* (0.00)	−0.00 (0.00)
Interest × age	−0.00*** (0.00)	−0.00*** (0.00)	−0.00** (0.00)	−0.00 (0.00)
Controls				
Female	−0.05*** (0.01)	−0.06* (0.02)	−0.00 (0.02)	−0.08** (0.02)
Education (ref. = 1: high school or less)				
Lower college	0.07*** (0.02)	0.08* (0.03)	0.05 (0.04)	0.12*** (0.03)
Bachelor's	0.15*** (0.02)	0.20*** (0.03)	0.08** (0.03)	0.20*** (0.03)
More than bachelor's	0.15*** (0.02)	0.24*** (0.04)	0.12* (0.05)	0.13*** (0.03)
Country (ref. = USA)				
UK	−0.06** (0.02)	–	–	–
France	0.01 (0.02)	–	–	–
Year (ref. = 2017)				
2019	0.34*** (0.01)	0.20*** (0.02)	0.33*** (0.02)	0.48*** (0.02)
*R*^2^ adjusted	0.38	0.44	0.36	0.37
*N*	8,947	3,017	2,945	2,985

*Note*. Estimates are unstandardized *b*-values with robust standard errors in parentheses. The model without an interaction term is available in the Supplementary Information file, Appendix C. The model in Appendix C includes unstandardized and standardized coefficients as well as exact *p*-values. OLS = ordinary least squares.

*p < .05, **p < .01, ***p < .001.

**H2:** The positive relationship between news consumption and online political expression will be stronger for younger (vs. older) respondents.

## Methods

This paper uses survey data gathered in three countries in 2017 (May to June, *N* = 4532) and 2019 (September to November, *N* = 4752). The data are pooled for analysis to ensure a sufficient sample size for estimating age-specific coefficients, then the year of data collection is added a statistical control with the values of 0 for data collection in 2017 and 1 for data collection in 2019. The sample is based on an online panel with quotas in place to ensure the age and sex representation of the population in each country. For the pooled sample, 50% are females and 50% are males. The country-specific sex quotas depend on the official statistics for each country. For example, our goal was 51% of the sample to be female in the United States, which we achieved; 49% for the United Kingdom, which we achieved; and, finally, 51% for France, where we achieved only 50%. In 2019, respondents could answer “non-binary,” but these responses (*n* = 15) were excluded from the analysis, leaving a balance of 9,284 respondents. The Supplementary Information file compares sex and age for the sample with the official sources for each country. To simplify this comparison, we created age categories. We were able to achieve our targets for young adults in the United States (12%) and the United Kingdom (11%), whereas in France we sought 10% of the sample in this age group and achieved 11%. In France, we had fewer people aged 55 or older in the sample than we wanted (goal: 42%, achieved: 40%).

In this paper, we include respondents ranging from age 18 to 75 years. We asked for the year of birth, then calculated age as the difference between birth year and the year of the survey, that is, 2017 or 2019 (*M* = 46.62, SD = 16.19).

The survey was administered by Lightspeed Kantar Group in both years and in all countries. The pooled sample includes 3,210 people from the United States, 3,043 from the United Kingdom, and 3,031 from France. The survey questions were identical in both studies. Given our choice of countries, we can significantly extend scholarship by studying France, which has rarely been studied as noted in recent meta-analyses and systematic reviews (Boulianne 2020; Rueß et al. 2019). Data were collected after ethics approval from MacEwan University (File Nos. 1617039 and 101662). Replication files are available at https://doi.org/10.6084/m9.figshare.16723054.

### Measures

For *online political expression*, we asked: “During the past 12 months, have you done any of the following online activities?” Respondents could choose from four answers: never, rarely, from time to time, or often. The list of activities included “sent political information to other people” (*M* = 1.72, SD = 0.96); “shared or posted political or campaign information via social media” (*M* = 1.60, SD = 0.92); “posted comments to political forums or blogs” (*M* = 1.50, SD = 0.87); and “commented on news websites” (*M* = 1.70, SD = 0.96). These four activities were combined into an additive index for online political expression ranging from 1 to 4 (Cronbach’s α = .89, *M* = 1.63, SD = 0.81).

The list of online activities continued, including “searched for political information online” (*M* = 2.26, SD = 1.05); “read stories on news websites” (*M* = 2.81, SD = 1.06); “read political forums or blogs” (*M* = 1.74, SD = 0.97); and “read political or campaign information via social media” (*M* = 1.92, SD = 1.02). These four activities were combined into an additive index for *online news* ranging from 1 to 4 (Cronbach's α = .82, *M* = 2.18, SD = 0.83). Country-specific averages and standard deviations are available in the Supplementary Information file.

For *political interest*, we asked: “How interested would you say you are in politics?” Respondents could choose from four answers: not at all interested, not very interested, fairly interested, and very interested (*M* = 2.74, SD = 0.96).

For *education*, the categories for each country were revised to offer a four-category classification. Approximately 47.13% of respondents have a high school diploma, 16.39% have a lower college education, 25.02% have a bachelor's degree, and 11.46% have a degree higher than a bachelor’s degree.

We also included a variety of statistical controls related to the study design, including the year of data collection (2017 or 2019) and country (United Kingdom, United States, and France).

### Analysis

We used Stata to conduct the analysis and create the figures. In terms of testing our hypotheses and research question, we made some assumptions about causal flow. The most contentious of these is the relationship between online news consumption and political interest—a relationship that may be reciprocal. For instance, Boulianne (2011) examines the causal flow between online news, political interest, and political talk (similar to political expression) using a three-wave panel of Americans. She finds that political interest at time 1 does not significantly predict online news consumption at wave 2; however, online news consumption at wave 2 predicts political interest at wave 3. Political talk was not correlated with online news consumption in any waves. Alternatively, Kruikemeier and Shehata (2017) focus on youth in Sweden, finding that political interest predicts online news consumption using their three-wave panel (also see Möller et al. 2018b; Shehata 2016). Thorson et al. (2018) also use a panel of American youth; they find strong significant positive relationships between wave 1 and wave 2 for political interest and political expression on social media. Bode et al. (2014) studied American youth and modeled news consumption, such as blogs and online news, as predictors of political uses of social media. While this body of research is inconclusive with different findings based on country and sample type, we opt for a model where political interest is treated as an antecedent of online news consumption (Table 1). Online news and political interest are independent variables in a model with political expression as a dependent variable (Table 2).

**Table 2.** Online News on Online Political Expression (OLS Estimates).

	All countries	United States	United Kingdom	France
Online news	1.04*** (0.02)	1.00*** (0.04)	1.09*** (0.04)	1.05*** (0.04)
Age (18–75 years)	0.01*** (0.00)	0.01*** (0.00)	0.01*** (0.00)	0.01*** (0.00)
Online news × age	−0.01*** (0.00)	−0.01*** (0.00)	−0.01*** (0.00)	−0.01*** (0.00)
Controls				
Female	−0.06*** (0.01)	−0.11*** (0.02)	−0.05* (0.02)	−0.02 (0.02)
Education (ref. = 1: high school or less)				
Lower college	0.02 (0.02)	−0.01 (0.03)	0.09** (0.03)	−0.00 (0.03)
Bachelor's	0.00 (0.01)	0.02 (0.03)	0.01 (0.02)	−0.02 (0.03)
More than bachelor's	−0.03 (02)	−0.01 (0.03)	−0.01 (0.04)	−0.07* (0.03)
Political interest (ref. = 1: not at all interested)				
Not very interested	−0.09*** (0.01)	−0.15*** (0.02)	−0.09*** (0.02)	−0.06** (0.02)
Fairly interested	−0.15*** (0.02)	−0.20*** (0.03)	−0.15*** (0.03)	−0.12*** (0.03)
Very interested	−0.03 (0.02)	0.01 (0.04)	−0.08* (0.04)	−0.04 (0.04)
Country (ref. = USA)				
UK	−0.08*** (0.01)	–	–	–
France	0.01 (0.01)	–	–	–
Year (ref. = 2017)				
2019	−0.08*** (0.01)	−0.08*** (0.02)	−0.07** (0.02)	−0.09*** (0.02)
*R*^2^ adjusted	0.59	0.62	0.55	0.57
N	8,940	3,016	2,944	2,980

*Note*. Estimates are unstandardized *b*-values with robust standard errors in parentheses. The model without an interaction term is available in the Supplementary Information file, Appendix D. The model in Appendix D includes unstandardized and standardized coefficients as well as exact *p*-values. OLS = ordinary least squares.

*p < .05, **p < .01, ***p < .001.

## Results

To address our research questions and hypotheses, the findings are presented in three steps. First, descriptive statistics speaking to age-related differences in online news use and online political expression across countries are presented (RQ1). Second, the role of political interest in explaining news use is studied—looking particularly at age differences in the relationship between political interest and online news use (H1). Third, we look more closely at the relationship between online news use and political expression, testing age differences in the relationship (H2).

### Age Differences in Online News Use and Online Political Expression

**Figure 1.**
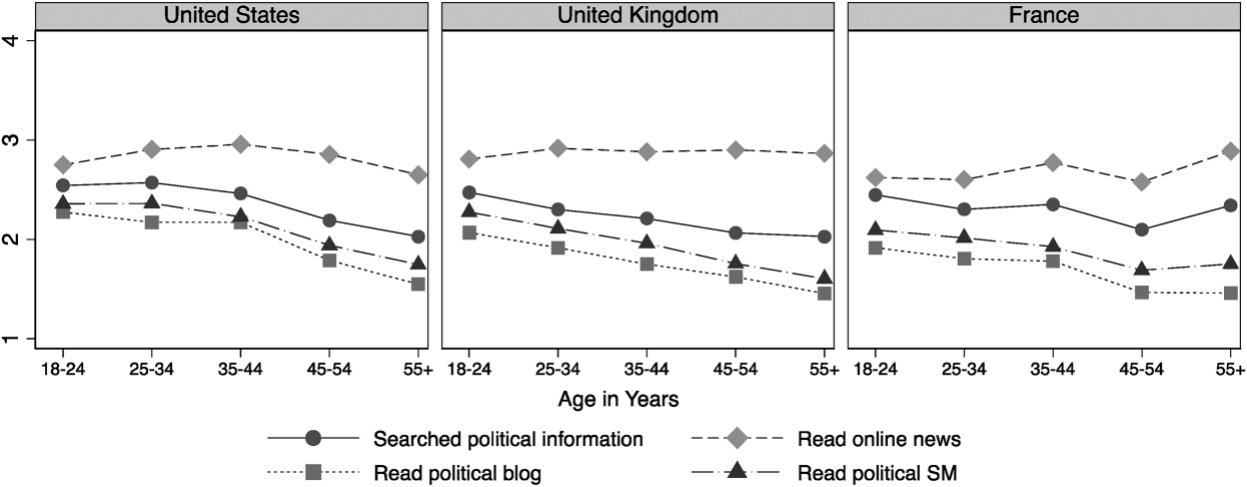
Use of online news by age (mean values).

*Note.* Lines represent mean levels of online news use. All variables range from 1 to 4. SM refers to social media. A minimum number of respondents: United States, *N* = 3,025; United Kingdom, *N* = 2,949; France, *N* = 2,987.

Figure 1 displays levels of online news use among different age groups, based on the separate indicators of news use (RQ1). A few patterns are worth noting. First, *reading stories on news websites* is the most common activity among all age groups, followed by *searching for political information*, *reading political or campaign information on social media*, and *reading political forums or blogs*. Second, most of these online activities are more frequent among younger citizens than older citizens. However, *reading stories on online news websites* appears to be the exception in this regard, with age being a less important factor across the three countries. Also, in France, reading online news is slightly more frequent among those aged 55 years or more compared to youth (18–24 years). Finally, the descriptive patterns noted are highly similar in the United States, United Kingdom, and France: young citizens are more frequent users of online news overall and the rank order of different news activities replicates cross-nationally.

**Figure 2.**
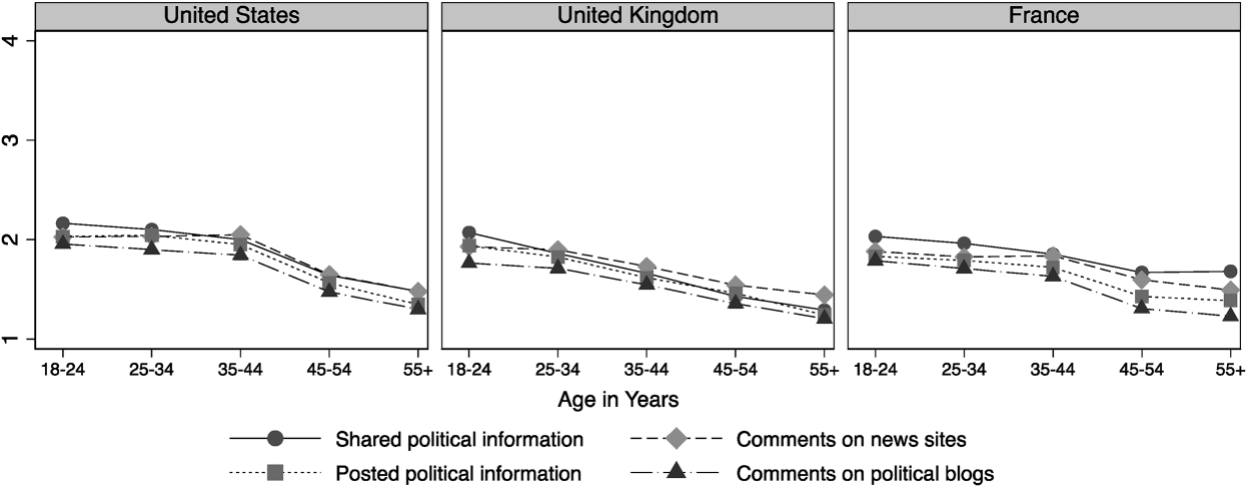
Online political expression by age (mean values).

*Note.* Lines represent mean levels of online political expression. All variables range from 1 to 4. A minimum number of respondents: United States, *N* = 3,024; United Kingdom, *N* = 2,949; France, *N* = 2,985.

Figure 2 illustrates similar descriptive data for online political expression. Generally, online political expression is less frequent than online news use. Differences between the four expression activities in terms of how frequently citizens engage in them are also less distinct. Again, however, clear age-related differences emerge in all three countries. Engaging in online political expression is still something that attracts younger citizens—and this holds true across the countries studied. The descriptive findings presented so far provide a few major surprises. Consuming news and engaging in political expression online are clearly related to age. What the data also show, however, is that these patterns are consistent across quite different national contexts.

### Political Interest on Online News Use

Turning to our hypotheses, we look more closely at age-related differences in the relationship between political interest and online media use (H1). Following our theoretical argument, we expected political interest to matter more for online news consumption among younger citizens than among older citizens. This hypothesis is tested in Table 1, which presents findings from four regression models—one pooled model as well as three country-specific models—predicting online news use. Here, we expect a positive relationship for political interest along with a negative interaction term with age. This is also what we see, with one exception. Political interest displays a positive coefficient in all countries, and the negative interaction term is statistically significant in the United States and the United Kingdom but not France.

The models predicting online news consumption also account for gender, education, country, and survey year (Table 1). Education is a positive and statistically significant predictor of online news consumption. Females are less frequent consumers of online news compared to males. Respondents from the UK are less frequent users of online news compared to respondents from the USA. Online news consumption was more frequent in 2019 than in 2017.

Returning to H1, Figure 3 displays the conditional relationship between political interest and online news for different age groups. Again, we see the expected positive correlation of political interest and online news across the board. This relationship is, however, significantly larger among young citizens. Findings from the pooled model show, for instance, that a 1-unit increase in political interest correlates with a 0.56 unit increase in news use in the youngest group (*b* =0.56, *p* < .001). The corresponding coefficient is only 0.40 in the oldest group (*b* = 0.40, *p* < .001). These age differences in the role of political interest are evident in the United States and the United Kingdom but not France.

### Online News Use on Online Political Expression

Turning to H2, we theorized that online news use should have a positive relationship with online political expression and, furthermore, that the correlation is stronger for younger respondents compared to older respondents. Table 2 presents findings from four regression models speaking to H2: one pooled model based on data from all three countries as well as three country-specific models. These models use the indexes for online political expression (dependent variable) and online news use (independent variable) as the key variables. The models use ordinary least squares (OLS) regression. An interaction term between online news and age is included to test the conditional relationship between news and online political expression (H2). Taken together, the findings are remarkably consistent across the three countries. In the pooled analyses, the stand-alone term for news use is positive and statistically significant (*b* = 1.04, *p* < .001). More importantly, the negative interaction term (*b* =  − 0.01, *p* < .001) indicates the relationship between news use on online political expression becomes weaker as age increases. These coefficients are almost identical when considering each country separately.

The models also control for gender, education, country, and survey year (Table 2). Education is not a statistically significant correlate of online political expression. Females are less engaged in online political expression compared to males. Respondents from the UK are less engaged in online political expression compared to respondents from the USA. Posting online was less frequent in 2019 than in 2017.

**Figure 4.**
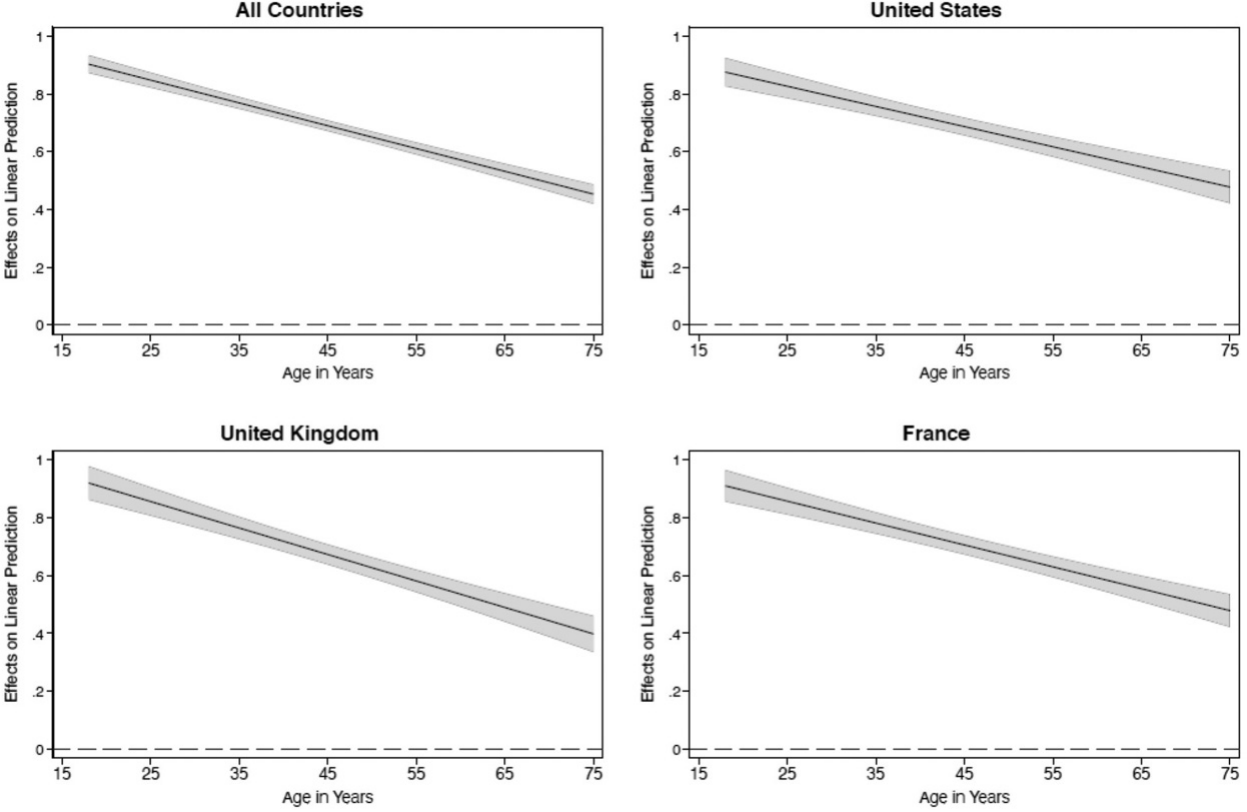
The marginal effect of news use on online political expression across different age groups.

*Note.* The marginal effect of online news use on online political expression and 95% confidence levels, based on the regression models in Table 2.

Returning to H2, Figure 4 illustrates the marginal effects of online news use on online political expression across age groups. Among the youngest respondents in our three samples, the relationship is clearly strongest: a 1-unit increase on the 1 to 4 news use scale corresponds to almost a 1-unit increase on the 1 to 4 political expression scale (*b* = 0.92, *p* < .001). But this coefficient is only half the size among the oldest respondents (*b* = 0.45, *p* < .001). The other graphs in the figure indicate this pattern is very similar in the three countries. Thus, while using online news has a positive and statistically significant relationship with online political expression across the board, the relationship is stronger for young citizens compared to older respondents.

## Discussion

This study establishes the growing importance of political interest as a personal motivation in current high-choice media environments (Prior 2007; Strömbäck et al. 2013; Thorson 2015) that political interest is a more important covariate of news consumption among young citizens compared to older generations (H1). Our findings based on data from three Western democracies reveal consistent cross-national support for the claim that political interest has a stronger positive relationship with online news consumption for young respondents compared to older respondents. Furthermore, online news consumption is more positively related to online political expression for younger respondents compared to older respondents (H2). While younger citizens may be motivated by political interest (Thorson 2015), older people may be more tied to a duty—duty to be informed and duty to participate—and this participation is in more institutionalized activities rather than the expressive activities enabled by social media platforms (Bennett 2008; Dalton 2008; Ohme 2019; Shehata et al. 2016). Importantly, however, we do not directly test citizenship norms and their role in news consumption and political activity. Contemporary research suggests asking about the importance of being informed and expressing oneself politically may not be the best way to capture the motivation to participate (Lane 2020).

Despite the robust scholarship discussing possible generational differences in citizenship norms, minimal research has tested age differences in citizenship norms. One exception is Ohme (2019), who establishes that older people are more likely to report dutiful and collective citizenship norms, that is, groups can change politics, compared to younger people in Denmark (see Table 3). However, neither of these types of norms predicted greater political participation. This body of research leaves many unanswered questions about citizenship norms, such as whether they differ by generation and if they are useful for understanding patterns of participation.

At the descriptive level, previous research has demonstrated a generational gap in online news consumption (Mangold et al. 2021; Newman et al. 2020; Scharkow et al. 2020; Taneja et al. 2018). However, we do not find large age differences in reading news websites in the three countries studied (see Figure 1; also see Scharkow et al. 2020)—although youth are more likely than older adults to read blogs and political information on social media. Patterns were also consistent with respect to our indicators of online political expression: the oldest age groups are the least likely to engage in online political expression. Let us briefly discuss three implications of our findings for research on political interest, changing modes of citizenship, and normative theories of democracy.

First, political interest is currently seen as the main personal motivation behind a range of outcomes related to news consumption, political knowledge, and participation more broadly (Andersen et al. 2020; Delli Carpini and Keeter 1996; Prior 2010). Prominent theories grounded in the OMA framework also suggest that political interest is becoming increasingly important with growing opportunities for media choice (Prior 2007; Strömbäck et al. 2013)—potentially widening gaps in news consumption, political knowledge, and participation. Put simply, with more choice opportunities available to citizens, personal motivations may become increasingly important for understanding differences in these outcomes. Our findings qualify and provide nuance to this argument. Political interest is clearly more important among some groups of citizens than others—even when their media environment is the same. This likely reflects generational differences in the persistence of early socialized media habits (Andersen et al. 2020; Ghersetti and Westlund 2018; Westlund and Weibull 2013). But this finding also challenges relatively widespread interpretations of the popular OMA framework: more choice opportunities *necessarily* translate into personal motivations becoming more important for understanding news media use. This seems to be more accurate among some groups of citizens than others—suggesting additional factors strongly related to age are at work.

Second, these differences may also reflect different modes of citizenship among younger and older generations. Even though AC and DC are less distinct in the real (empirical) world than suggested by these conceptualizations (Bennett 2008; Dalton 2008; Shehata et al. 2016), the greater reliance on political interest as an intrinsic personal motivation for news consumption among young citizens suggests some generational differences in modes of citizenship. The fact that our findings are very similar across countries strengthens the robustness and universality of these claims. At the same time, our results are only indicative in this regard. Whether the patterns actually reflect generational differences in modes of citizenship that will persist over time, or whether they reflect life-cycle effects that disappear as young citizens grow older, cannot be determined from the data presented here. Thus, while we follow Andersen et al. (2020) in explaining these age-specific patterns as a cohort effect, time series analysis is needed to evaluate whether these distinct forms of news consumption and participation extend into the life cycle. In this paper, we did not label the distinct generations, for example, Gen X or Gen Z, or match up the data to this generational discourse. Additional research could consider whether these generational distinctions are appropriate in a cross-national perspective, as existing research tends to use single-country surveys to mark generations. Notably, our data did not reveal distinct breaks in patterns of digital media use or online political expression (see Figures 1 and 2) based on age. The patterns were quite linear, which runs contrary to the assumptions related to naming distinct generations.

Third, our findings also have normative implications. Any normative assessments will, however, depend on what normative theory of democracy serves as the point of reference (Althaus 2012; Teorell 2006). While personalized forms of news consumption and online political engagement may strengthen certain democratic values related to political freedom, pluralism, and participatory ideals, large gaps in participation driven by individual motivation and differences in political interest may also threaten democratic values related to political equality and equal representation. Thus, if the age differences documented here presage a future era where political interest becomes increasingly important for democratic citizenship, these developments will be evaluated rather differently depending on the normative model of democracy to which one adheres. While pluralistic and some forms of participatory theories of democracy may praise the good sides of increasing opportunities for citizen engagement, growing gaps may be more problematic for theories focusing on representative democracy, electoral participation, and political equality (Andersen et al. 2020; Delli Carpini and Keeter 1996; Teorell 2006).

Finally, we highlight a few key limitations of our study. To begin with, the findings presented here are based on cross-sectional data and, thus, cannot untangle the causal direction of political interest, online news consumption, and online political expression. Panel studies suggest differences in results based on samples of adults versus youth and based on countries (see prior discussion of Boulianne 2011; Kruikemeier and Shehata 2017; Möller et al. 2018a; Shehata 2016; Thorson et al. 2018). Our assumption, grounded in theory as well as previous studies, is that political interest drives news consumption. However, mutual influences and reciprocal relationships are also highly likely. Furthermore, many factors influence the connection between news consumption and political expression on social media. Kümpel (2020) offers a list of considerations including characteristics of the news provider, the content, news curator, news recommendation, and news receivers. Further research might explore these factors in understanding age differences in patterns of news consumption and engagement.

In addition, we have data on only three countries, all of which are advanced Western democracies. As such, we do not know the age patterns in political interest, online news consumption, and online political expression beyond these countries. A meta-analysis of research comparing free press and lack of free press systems suggests the relationship between social media news consumption and political participation differs in these two types of systems (Boulianne 2019). As mentioned, Toff and Kalogeropoulos (2020: 367) describe our three countries (and others) as “cultures of news consumption,” given their high degree of press freedom and political stability. The study of youth, digital media, and political participation is biased toward these types of countries (Boulianne and Theocharis 2020). Future research should examine whether generational differences in political interest, online news consumption, and political expression on social media extend beyond Western democracies and to systems without a free press.

## Supplemental Material

sj-docx-1-ijpp-10.1177_19401612211060271 - Supplemental material for Age Differences in Online News Consumption and Online Political Expression in the United States, United Kingdom, and France Click here for additional data file. Supplemental material, sj-docx-1-ijpp-10.1177_19401612211060271 for Age Differences in Online News Consumption and Online Political Expression in the United States, United Kingdom, and France by Shelley Boulianne and Adam Shehata in The International Journal of Press/Politics
