# Supplementary material for: Age Differences in Online News Consumption and Online Political Expression in the United States, United Kingdom, and France
Source: Int J Press Polit. 2021 Dec 20;27(3):763–83. doi: 10.1177/19401612211060271 (PMC9058403; doi:10.1177/19401612211060271)
Supplement: sj-docx-1-ijpp-10.1177_19401612211060271 - Supplemental material for Age Differences in Online News Consumption and Online Political Expression in the United States, United Kingdom, and France [file sj-docx-1-ijpp-10.1177_19401612211060271.docx]

Age Differences in Online News Consumption and Online Political Expression

in the United States, United Kingdom, and France

Shelley Boulianne and Adam Shehata

*International Journal of Press/Politics*

Replication files: <https://doi.org/10.6084/m9.figshare.16723054>

**Supplementary Information**

*Appendix Table A: Country samples*

| Age |  | USA | UK | France |
| --- | --- | --- | --- | --- |
| 1 18-24 | Official  Survey | 12%  12% | 11%  11% | 10%  11% |
| 2 25-34 | Official  Survey | 18%  18% | 17%  17% | 15%  16% |
| 3 35-44 | Official  Survey | 16%  17% | 16%  16% | 16%  16% |
| 4 45-54 | Official  Survey | 17%  17% | 18%  18% | 17%  17% |
| 5 55+ | Official  Survey | 37%  36% | 37%  38% | 42%  40% |
| Sex |  | USA | UK | France |
| 0 Male | Official  Survey | 49%  49% | 51%  51% | 49%  50% |
| 1 Female | Official  Survey | 51%  51% | 49%  49% | 51%  50% |

U.S. Official Source

Age and sex (2017): Age in Entire U.S. for 2017 American Community Survey

U.K. Official Source

Sex and age (2016):

<https://www.ons.gov.uk/peoplepopulationandcommunity/populationandmigration/populationestimates/bulletins/annualmidyearpopulationestimates/mid2016#main-points>

France:

Age and sex (2018):

<https://www.insee.fr/en/statistiques/2382609?sommaire=2382613>

*Appendix Table B:* *Means, standard deviations, and percentages for each country (ages 18 to 75 years)*

|  | United States | United Kingdom | France |
| --- | --- | --- | --- |
| Sent/Shared political information to other people online | 1.80 (1.01) | 1.57 (0.88) | 1.79 (0.95) |
| Shared or posted political or campaign information via social media | 1.71 (1.00) | 1.52 (0.89) | 1.56 (0.87) |
| Commented on news website | 1.78 (1.01) | 1.64 (0.93) | 1.66 (0.93) |
| Posted comments to political forums or blogs | 1.62 (0.95) | 1.44 (0.80) | 1.44 (0.82) |
| Searched for political information online | 2.30 (1.08) | 2.16 (1.02) | 2.31 (1.05) |
| Read stories on news website | 2.80 (1.07) | 2.88 (1.03) | 2.74 (1.06) |
| Read political forums or blogs | 1.91 (1.05) | 1.69 (0.93) | 1.62 (0.91) |
| Read political or campaign information on social media | 2.06 (1.07) | 1.86 (0.99) | 1.85 (1.00) |
| Political interest | 2.82 (0.98) | 2.78 (0.90) | 2.62 (0.99) |
| Education | 2.22 (1.08) | 1.88 (1.05) | 1.91 (1.10) |
| Age (18-75) | 45.57 (16.20) | 46.82 (16.29) | 47.50 (16.02) |
| Female | 51.28% | 49.66% | 50.39% |

*Appendix Table C:* *Political interest on online news (OLS estimates), without interaction term; with standardized estimates*

|  | b | SE | B | p |
| --- | --- | --- | --- | --- |
| All countries |  |  |  |  |
| Political interest | 0.483 | 0.01 | 0.562 | < .001 |
| Age (18-75) | -0.011 | 0.00 | -0.218 | < .001 |
| Female | -0.054 | 0.01 | -0.032 | < .001 |
| Lower college | 0.071 | 0.02 | 0.032 | < .001 |
| Bachelor’s | 0.148 | 0.02 | 0.078 | < .001 |
| More than bachelor’s | 0.144 | 0.02 | 0.055 | < .001 |
| UK | -0.061 | 0.02 | -0.035 | < .001 |
| France | 0.011 | 0.02 | 0.006 | .504 |
| 2019 | 0.341 | 0.01 | 0.206 | < .001 |
| R^2^ adjusted | 0.38 |  |  |  |
| N | 8,947 |  |  |  |
| United States |  |  |  |  |
| Political interest | 0.524 | 0.01 | 0.581 | < .001 |
| Age (18-75) | -0.016 | 0.00 | -0.285 | < .001 |
| Female | -0.061 | 0.03 | -0.035 | .013 |
| Lower college | 0.078 | 0.03 | 0.035 | .022 |
| Bachelor’s | 0.193 | 0.03 | 0.100 | < .001 |
| More than bachelor’s | 0.237 | 0.04 | 0.092 | < .001 |
| 2019 | 0.202 | 0.02 | 0.114 | < .001 |
| R^2^ adjusted | 0.44 |  |  |  |
| N | 3,017 |  |  |  |
| United Kingdom |  |  |  |  |
| Political interest | 0.483 | 0.01 | 0.557 | < .001 |
| Age (18-75) | -0.012 | 0.00 | -0.256 | < .001 |
| Female | -0.004 | 0.02 | -0.003 | .859 |
| Lower college | 0.052 | 0.04 | 0.020 | .184 |
| Bachelor’s | 0.079 | 0.03 | 0.046 | .004 |
| More than bachelor’s | 0.118 | 0.05 | 0.039 | .010 |
| 2019 | 0.330 | 0.02 | 0.211 | < .001 |
| R^2^ adjusted | 0.36 |  |  |  |
| N | 2,945 |  |  |  |
| France |  |  |  |  |
| Political interest | 0.448 | 0.01 | 0.554 | < .001 |
| Age (18-75) | -0.005 | 0.00 | -0.108 | < .001 |
| Female | -0.077 | 0.02 | -0.048 | .001 |
| Lower college | 0.118 | 0.03 | 0.057 | < .001 |
| Bachelor’s | 0.200 | 0.03 | 0.093 | < .001 |
| More than bachelor’s | 0.130 | 0.04 | 0.055 | < .001 |
| 2019 | 0.480 | 0.02 | 0.300 | < .001 |
| R^2^ adjusted | 0.37 |  |  |  |
| N | 2,985 |  |  |  |

*Males, high school or less, USA, and 2017 data collection are the reference groups for the above analysis.

*Appendix Table D:* *Online news on online political expression (OLS estimates), without interaction term; with standardized estimates*

|  | b | SE | B | p |
| --- | --- | --- | --- | --- |
| All countries |  |  |  |  |
| Online News | 0.686 | 0.01 | 0.704 | < .001 |
| Age (18-75) | -0.008 | 0.00 | -0.165 | < .001 |
| Female | -0.060 | 0.01 | -0.037 | < .001 |
| Lower college | 0.009 | 0.02 | 0.004 | .573 |
| Bachelor’s | -0.006 | 0.01 | -0.003 | .696 |
| More than bachelor’s | -0.041 | 0.02 | -0.016 | .031 |
| Not very interested | -0.065 | 0.02 | -0.035 | .001 |
| Fairly interested | -0.123 | 0.02 | -0.075 | < .001 |
| Very interested | -0.009 | 0.02 | -0.005 | .718 |
| UK | -0.087 | 0.01 | -0.051 | < .001 |
| France | -0.012 | 0.01 | -0.007 | .393 |
| 2019 | -0.082 | 0.01 | -0.051 | < .001 |
| R^2^ adjusted | 0.57 |  |  |  |
| N | 8,940 |  |  |  |
| United States |  |  |  |  |
| Online News | 0.691 | 0.02 | 0.699 | < .001 |
| Age (18-75) | -0.007 | 0.00 | -0.132 | < .001 |
| Female | -0.114 | 0.02 | -0.065 | < .001 |
| Lower college | -0.010 | 0.03 | -0.005 | .718 |
| Bachelor’s | 0.012 | 0.03 | 0.007 | .623 |
| More than bachelor’s | -0.016 | 0.03 | -0.006 | .626 |
| Not very interested | -0.124 | 0.04 | -0.056 | .001 |
| Fairly interested | -0.168 | 0.04 | -0.093 | < .001 |
| Very interested | 0.034 | 0.04 | 0.017 | 0.420 |
| 2019 | -0.086 | 0.02 | -0.049 | < .001 |
| R^2^ adjusted | 0.61 |  |  |  |
| N | 3,016 |  |  |  |

*Males, high school or less, not at all interested in politics, USA, and 2017 data collection are the reference groups for the above analysis.

*Appendix Table D:* *Online news on online political expression (OLS estimates), without interaction term; with standardized estimates continued*

|  | B | SE | B | p |
| --- | --- | --- | --- | --- |
| United Kingdom |  |  |  |  |
| Online News | 0.665 | 0.02 | 0.685 | < .001 |
| Age (18-75) | -0.008 | 0.00 | -0.177 | < .001 |
| Female | -0.044 | 0.02 | -0.029 | .030 |
| Lower college | 0.088 | 0.03 | 0.035 | .007 |
| Bachelor’s | -0.000 | 0.02 | 0.000 | .985 |
| More than bachelor’s | -0.030 | 0.04 | -0.010 | .428 |
| Not very interested | -0.059 | 0.04 | -0.032 | .104 |
| Fairly interested | -0.112 | 0.04 | -0.074 | .002 |
| Very interested | -0.059 | 0.04 | -0.032 | .173 |
| 2019 | -0.077 | 0.02 | -0.051 | < .001 |
| R^2^ adjusted | 0.53 |  |  |  |
| N | 2,944 |  |  |  |
| France |  |  |  |  |
| Online News | 0.693 | 0.02 | 0.728 | < .001 |
| Age (18-75) | -0.009 | 0.00 | -0.187 | < .001 |
| Female | -0.023 | 0.02 | -0.015 | .224 |
| Lower college | -0.009 | 0.03 | -0.005 | .716 |
| Bachelor’s | -0.037 | 0.03 | -0.018 | .175 |
| More than bachelor’s | -0.083 | 0.03 | -0.037 | .005 |
| Not very interested | -0.029 | 0.03 | -0.018 | .342 |
| Fairly interested | -0.092 | 0.03 | -0.056 | .006 |
| Very interested | -0.019 | 0.04 | -0.010 | .630 |
| 2019 | -0.090 | 0.02 | -0.059 | < .001 |
| R^2^ adjusted | 0.56 |  |  |  |
| N | 2,980 |  |  |  |

*Males, high school or less, not at all interested in politics, USA, and 2017 data collection are the reference groups for the above analysis.
